# Development of Motor Imagery in School-Aged Children with Autism Spectrum Disorder: A Longitudinal Study

**DOI:** 10.3390/brainsci12101307

**Published:** 2022-09-28

**Authors:** Anna-Maria Johansson, Thomas Rudolfsson, Anna Bäckström, Louise Rönnqvist, Claes von Hofsten, Kerstin Rosander, Erik Domellöf

**Affiliations:** 1 Faculty of Social Sciences, Department of Psychology, Umeå University, 90187 Umeå, Sweden; 2 Centre for Musculoskeletal Research, Department of Occupational Health Sciences and Psychology, University of Gävle, 80176 Gävle, Sweden; 3 Department of Psychology, Uppsala University, Box 1225, 75142 Uppsala, Sweden

**Keywords:** autism spectrum disorder, longitudinal, motor imagery, visual imagery, development

## Abstract

Autism spectrum disorder (ASD) is a diagnosis based on social communication deficits and prevalence of repetitive stereotyped behaviors, but sensorimotor disturbances are commonly exhibited. This longitudinal study aimed at exploring the development of the ability to form mental motor representations (motor imagery; MI) in 14 children with ASD and 17 typically developing (TD) children at 7, 8 and 9 years of age. MI was investigated using a hand laterality paradigm from which response times (RT) and error rates were extracted and compared with performance on a visually based mental rotation task (VI). A criterion task was used to ensure that the children could perform the task. The results showed wide performance variability in the ASD group with more failures than TD in the MI criterion task, especially at 7 years. For all age levels and both the MI and VI tasks, the error rates were significantly higher and RTs longer for the ASD group compared with TD. Signs of MI strategies were however noted in the ASD group as biomechanically constrained orientations had longer RTs than less constrained orientations, a RT pattern that differed from the VI task. The presence of MI in the ASD group was most evident at 9 years, but the error rates remained high at all ages, both in the MI and VI task. In comparison, the TD group showed stable MI strategies at all ages. These findings indicate that MI ability is delayed and/or impaired in children with ASD which may be related to difficulties performing required mental rotations.

## 1. Introduction

Autism spectrum disorder (ASD) is a life-long clinically heterogeneous neurodevelopmental disorder with specific impairments in social communication and repetitive stereotyped behaviors and interests [1]. Although not part of the diagnostic features, the prevalence of motor difficulties in children with ASD has been reported at 80–90% [2,3] with evident associations to the core symptomology [4] and intellectual functioning [3]. Consequently, motor difficulties appear to be implicated in a wide range of behaviors and functions in children with ASD. Still, to date, there is no consistent theory explaining the basis for these difficulties. Researchers have, however, investigated several different aspects hypothesized to influence movement proficiency including visuomotor adaptation [5]; procedural learning [6]; motor memory [7]; multisensory feedback mechanisms [8]; executive processes [9] and intellectual functioning [10].

A promising avenue of investigation in this context is the study of pre-motor processes in movement control, as insufficient planning or programming of movements could lead to a wide range of performance disturbances [11,12] including gross motor functions and goal-directed movements with object interaction. Insufficient planning and programming of movements could also be manifested as a poorer ability to make sufficient on-line corrections when needed during task execution [13,14,15]. This could be due to corrective actions plans being flawed and general overarching action plans poorly formulated. It has been proposed that such prospective (or predictive) control deficits may contribute to the movement deficits observed in ASD [16,17,18]. Investigations into the functions of prospective control mechanisms of the motor system in ASD have been operationalized through measurements of different movement kinematics and the coordination between hand and eye movements during actual performance [6,19,20]. The results suggest that there are deviances in ASD with regard to the orchestration of multiple action plans into precise overall planning and the following execution of action sequences [6,20]. Further, reductions in motor anticipation under conditions of uncertainty, a frequent aspect in social interaction as this requires coordination with the other agent which actions cannot be fully predicted, have been shown [19]. It has been suggested that predictive processing deficits may underlie some of the core symptoms of ASD including insistence on sameness and deficits in social interaction [21,22]. Cannon et al. [22] suggests that social prediction differences in ASD could be the most notable expression of an underlying domain general predictive processing deviance.

Predictive motor processes can also be investigated through assessments of motor imagery (MI) ability. MI is an implicit simulation process where an internal model is generated and used in prediction of the consequences of an action [12]. Embedded in the concept of MI is that entailed processes are predictive in nature, represented as action goals that do not directly correspond to the current state of the world. Instead, our representation of action is related to what the state of the world will be if the action is executed. In relation to movement control, an inability to engage in MI would lead to slow and inept actions, relying mainly on sensory feedback for modulation and correction. Behavioral evidence suggest that MI is modulated by similar constraints as those applied on the body during overt actions, for example, the duration of actual and imagined movements are similar and can be modified by an imagined load to carry [23]. Furthermore, neuroimaging studies show that MI share neural correlates with motor actions [14].

MI ability has been studied in paradigms where mental own-body transformations in space are required. One such test is the hand laterality judgement (HLJ) task, where judgements of whether a right or left hand is depicted, typically on a computer screen. The task is solved by mentally applying the own body into the position of the hand. Response times (RTs) are reflected as the time it takes to mentally simulate this own-body transformation. The hand stimulus is oriented in different orientations where some orientations result in biomechanically challenging positions. The RTs of orientations with high load on biomechanical constraints are thus longer than those with a low load. Hence, a biomechanical effect can be seen in typically developing populations [24]. The biomechanical effect is commonly compared with a visually based mental orientation task that is non-corporeal (i.e., objects, numbers or letters that are not related to transformation of a body part; visual imagery; VI). By contrasting the corporeal and non-corporeal sources of information a better knowledge of the mental orientation strategies used in the tests can be achieved.

Regarding the development of MI during childhood, the majority of typically developing children between 5 and 12 years of age have an adult-like performance on versions of the HLJ task, although with fewer correct responses [25]. Others [26] show stronger biomechanical effects and increased own-body movements to solve the task in younger children (6 years old) compared to older ones (7–10 years old). At 7–8 years of age, there seems to be a transitional period where laterality judgements primarily are made mentally, i.e., without accompanying overt movements [27]. However, outcomes for young children are dependent on the nature of the MI task where more implicit designs without direct instructions to use MI to solve the task (as used in the current study) show presence of MI at younger ages than tasks that have a more explicit nature [25]. From 11 to 12 years of age, further refinement of MI has been observed across stages of adolescence, with the strongest biomechanical effect not evident until late adolescence [27]. To date, a limited number of studies have investigated MI ability in children or adolescents with ASD [28,29,30,31,32]. Results from these studies are mixed. Some studies report absence of a biomechanical effect in children and adolescents with ASD on tasks where mental activation of sensorimotor information during mental imagery of actions is required [28,31] while others show present but altered MI ability [29,30,32].

The study by Conson et al. [32] used a HLJ paradigm and results showed biomechanical effects in both the ASD and control group (age range 10–20 years), thus implicating that MI processes were used. It was however also evident that MI was influenced by concurrent body posture within the ASD group to a greater extent than in controls. This was taken as evidence for immaturity in the development of, or lack of efficiency in, decoupling body position from mental body transformations. The participants with ASD also showed higher error rates compared to the typically developing control group, indicating greater difficulty with performing MI. However, the age span was quite large and MI might follow different developmental trajectories in ASD and typically developing children. The HLJ paradigm was also used by Chen and colleagues [29] where MI was investigated in 11–15-year-old children with ASD. They found functional but inefficient MI ability in the ASD group as a biomechanical effect was apparent in the RTs, but responses were significantly slower than in the control group. This was further compared with a visual (non-corporeal) mental orientation task where no group differences in RTs were evident. The difference in error rate between the groups was not significant, indicating preserved accuracy in the ASD sample. Conson et al. [31] have also used a paradigm where hand laterality was judged (as in HLJ) in 12–16-year-old children with ASD. They reported atypical performance in the ASD group with signs of visually based mental orientation strategies and a lack of MI in the ASD group. Taken together, outcomes from MI studies are mixed and to date no longitudinal studies investigating developmental change in MI ability of children with ASD are known to us.

The present longitudinal study investigated the development of MI ability by extracting RTs and error rates on a MI- and a VI task at three age levels (7, 8 and 9 years of age) in a sample of children with ASD compared to a typically developing (TD) control group. The utilization of a MI strategy was expected to be shown if RTs were higher for the more biomechanically challenging lateral position than the less biomechanically challenging medial position. We specifically wanted to determine if MI is a strategy used by ASD children compared with TD controls, and how MI ability may change with development across the ages studied. An additional objective was to explore MI and VI performance patterns over age. To further deepen the understanding of performance we also investigated associated error rates.

## 2. Materials and Methods

This study was a longitudinal design with three measurement ages, one year apart including both children with ASD and a TD control group. The study was approved by the regional ethical board and conducted in accordance with the Declaration of Helsinki.

### 2.1. Participants

Fourteen children with ASD (4 girls; M age; 7 years 10 months; 5 left-handed) and 17 TD children (9 girls; M age; 7 years 9 months; all right handed) were initially recruited to participate at age level 1 (age level 1; A1). With one-year intervals, the children were re-invited twice (age level 2 and 3; A2 and A3) for follow up tests (A2 ASD; n = 12; *M* age ASD = 8 years 10 months; A3 ASD; n = 12, *M* age = 9 years 11 month: A2 TD; n = 17; *M* age = 8 years 9 months; A3 TD; n = 17, *M* age = 9 years 9 months). The two dropouts between A1 and A2 in the ASD group were boys, 1 right- and 1 left-handed. There were no significant age differences between the groups at any age level as determined by independent t-tests. All children were deemed to have a fluid intelligence index within the normal range as determined by Wechsler Intelligence Scale for Children, 5th Ed. [33]. (WISC-V; Table 1). The children with ASD were initially recruited from the local child habilitation center and the TD children were recruited from local schools and by convenience sampling. All children gave their assent to participate in the study and the guardians of the children signed an informed consent form prior to participation.

### 2.2. Experimental Setup

Two tasks were used; a HLJ task engaging MI and a non-corporeal visual imagery (VI) task (displaying the number “2”) where visually based mental rotations were sufficient for solving the task. The MI stimulus depicted back of hands and was selected as it has been shown to be less influenced by IQ and easier for young children to perform than palmar view stimuli [34]. Furthermore, the palmar view does not seem to better extract the biomechanical effect in children below 10 years of age compared to back hand stimuli [35]. In the MI and VI tasks, children were asked to determine if they saw a right or left hand or a correct or mirror-reversed number 2 on a CRT computer screen (100 Hz refresh rate) by pressing a hand-held response button (millisecond accurate USB- response button; www.blackboxtoolkit.com). One response button was placed in each hand. The children had to respond whether it was a right or a left hand depicted on the screen for the MI task by pressing the button in their corresponding hand. For the VI task, the instruction was to press the button in the right hand if the number 2 was depicted in the correct way and the button in the left hand if it was mirrored reversed. The hands of the children were occluded during the experiment to minimize the risk of the effect of posture manipulation to fit the stimuli orientation [32,34] and potential visual feedback from looking at their own hands [34]. The children were instructed that it was of equal importance to be as quick as possible and to give the correct answer. Not all the children knew the difference between right and left so all were instructed to respond by pressing the response button of the hand that corresponded to the one depicted on the screen.

**Figure 1:**
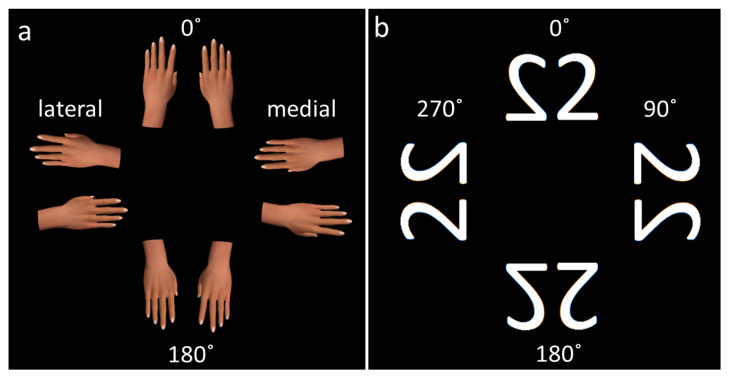
Stimuli used in the (**a**) MI task (courtesy of [34]) and (**b**) VI task at the different orientations. Hand images in the lateral orientation were regarded as stimuli with high biomechanical constraint and those in the medial orientation as low biomechanical constraint.

The experiment (run in OpenSesame, https://osdoc.cogsci.nl/, accessed on 10 September 2018) started with a count down from five after which a white centered fixation cross was shown on a black background. This was followed by the presentation of a brief (500 ms) black background and then the experimental stimulus was displayed, either a hand or the number 2, also in a centered position and on a black background. The experimental stimuli were displayed until a response was recorded. After the recorded response a black background was displayed for 500 ms. This was followed by the white fixation cross (displayed at varied durations between 509 and 1499 ms) and then the black screen for 500 ms. The hand image was in color and the number two was white (Figure 1).

Both the MI and the VI task consisted of a criterion task, a practice trial and the experimental trial given in that order. Only outcomes from the experimental trials were used in the analyses. The MI criterion task contained ten back hand images (presented one at a time) with zero orientation, five right- and five left-hand images, where a minimum of six correct answers were required to proceed to the practice trial and the experimental trial. The requirement of six correct answers set to ensure performance above chance level (following a binominal distribution). In the MI practice trial, six hand images, a mix of right and left hands, were displayed encompassing the experimental orientations (0°, 90°, 180° and 270° rotations). During the MI practice trial, the experimenter gave feedback to the participant if their responses were correct or not to ensure that the child had understood the task. The MI experimental trial consisted of 24 hand images; 12 right and 12 left hands at the four different orientations (each hand stimuli being presented three times in total). No feedback was given from the experimenter during the MI experimental trial. The VI task followed the same set up; a criterion task, a practice trial and the experimental trial. During the practice trial the experimenter gave feedback to the children’s responses as in the MI task.

### 2.3. Outcome Variables

Response times were extracted in ms from the onset of the stimulus to the button was pressed. The responses were also recorded as correct or incorrect. For the MI task, hand outcomes were organized into; 0°, 180°, medial, and lateral orientations in accordance with the procedure applied by Spruijt and colleagues [34]. Likewise, RTs in the VI task were also coded according to orientation (0°, 90°, 180°, and 270° rotation) as experimental conditions.

### 2.4. Statistics

#### 2.4.1. Response Times

A series of linear mixed models were used to target the research questions related to RTs (IBM SPSS, Statistics, Version 27 [36]). For longitudinal analyses the RTs were averaged for each orientation on the individual level. In these models’ participant ID and orientation (categorical variables) were specified as subjects and age level as repeated measures. An unstructured covariance matrix was used. Participant ID was specified as a random factor with a unique intercept. Age level, orientation and group were used as fixed effects main effects, and interactions between age level × group, and group × orientation were specified. In cross-sectional models raw RTs were used. In these analyses participant ID was specified as subject and orientation angle as repeated measures. The covariance matrix used was unstructured. Again, participant ID was specified as a random factor with a unique intercept. Orientation, task and group were used as fixed effects and main effects, and interactions between group × task and orientation × task were specified. These models were stratified by age level. Sidak correction was used to correct for multiple comparisons in all models. The distributions of RT’s were skewed and thus transformed by the natural logarithm. For all significant effects, the geometrical mean difference was calculated to convert the logarithmic RTs to an understandable quantity. Estimates of fixed effects was reported as the difference in geometrical mean (*GMD*) derived from the inverse logarithm of the parameter estimates and the confidence interval limits.

#### 2.4.2. Error Rates

Mixed effects logistic regression with random intercept for participant ID was used to analyze error rates in the MI and VI tasks (Jamovi [37], GAMLj: General analyses for linear models [38]). A full model (group × age level × orientation) was used to identify main effects in the likelihood of making an incorrect answer. Further, models stratified by group was used to test within group differences (age level × orientation) and models stratified by age level (group × orientation) was used to test the group and orientation differences at the different ages. TD, A1 and orientation 0° were used as references in these analyses. Group effects were presented as the odds ratio (OR) with the TD group as reference.

## 3. Results

### 3.1. Criterion Tasks

All children in the TD group passed the MI and VI criterion tasks at all age levels. In contrast, a substantial number of children from the ASD group did not pass the MI criterion task, especially prominent at 7 years (A1 n = 5; A2 n = 4; A3 n = 2). Three of the children who failed the criterion task at A1 also failed at A2. One child failed the MI criterion task at all age levels. Pass rate was higher for the children with ASD in the VI criterion task (A1 n = 2; A2 n = 2). All of these children also failed the MI criterion task at one or more age levels. Of the five children who failed the MI criterion task at 7 years one also failed the VI criterion task at the same age level. These children had neither the lowest nor the highest scores on the WISC-V test.

### 3.2. Motor Imagery Task Outcomes

#### 3.2.1. Response Times

**Figure 2:**
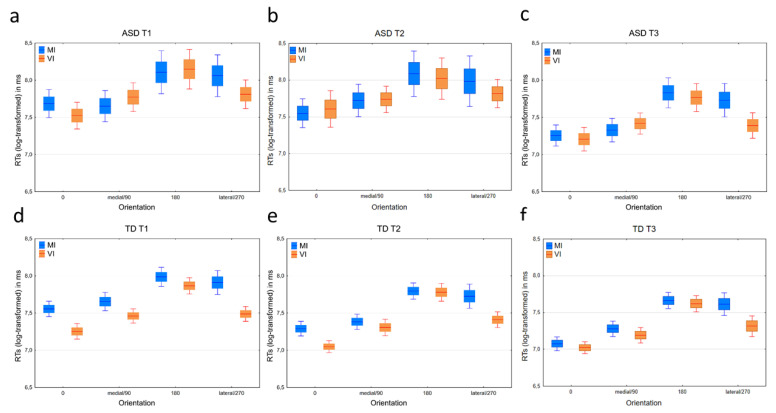
RTs (log-transformed) in separate graphs for the different orientations and tests by the age levels (A1 (**a**) and (**d**); A2 (**b**) and (**e**); and A3 (**c**) and (**f**)) and groups (ASD (**a**–**c**) and TD (**d**–**f**)). Means are indicated by the lines, standard errors by the box and 95% confidence intervals by the whiskers.

While the *GMD* was 17% higher in the ASD group than in the TD group, no Group effect was detected (Table 2) reflecting longer RTs for the ASD group. A main effect of age was shown where all compared age levels differed from one another with a general reduction in *GMD*s with increasing age, reflecting reduced RTs over time. Similarly, a significant effect was evident for orientation with the 180° orientation differing the most from 0° orientation. Importantly, the medial orientation had lower *GMD*s than the lateral orientation suggesting the presence of a general biomechanical effect. There were no significant interaction effects between group × age level nor group × orientation (see Table 2 for specifics; Figure 2a–d).

#### 3.2.2. Error Rates

The mixed effects logistic regression with random intercept for participant ID showed that group (χ^2^ = 16.5, 1, OR = 7.37; [CI = 2.81–19.31] *p* < 0.001) and age level (χ^2^ = 15.1, 2, *p* < 0.001, OR A2–A1 = 1.24 [95% CI = 0.86–1.78]; OR A3–A1 = 0.58 [95% CI = 0.39–0.87]) and orientation (χ^2^ = 53.4, 3, *p* < 0.001, OR medial-0° = 1.94 [95% CI = 1.18–3.18], OR 180°-0° = 3.94 [95% CI = 2.47–6.31], OR lateral-0° = 4.70 [95% CI = 2.95–7.49]) were significant predictors of making incorrect answers.

##### Effects of Age

Follow-up analyses of the effects of group and orientation stratified by age showed that the ASD group were significantly more likely to make errors compared to the TD group at all three age levels (A1 χ^2^ = 5.38, 1, *p* = 0.02; OR = 7.59, [95% CI OR = 1.37–42.05], *p* = 0.02; A2 χ^2^ = 7.64, 1, *p* = 0.006, OR = 6.0 [95% CI = 1.68–21.36]; A3 χ^2^ = 11.2, OR = 13.31 [95% CI = 2.91–60.71] *p* = 0.001). At A1, orientation was significant (χ^2^ = 19.95, 3, *p* = 0.001) as a main effect. Sublevel effects were significant in the lateral-0° contrast (OR = 5.21 [95% CI = 2.33–11.61] *p* < 0.001) and the 180°-0° contrast (OR = 3.25 [95% CI = 1.44–7.30] *p* < 0.004). The medial-0° contrast was non-significant (*p* = 0.29). At A2, orientation was again significant (χ^2^ = 23.84, 3, *p* = 0.001) as a main effect and the likelihood of making errors were significantly increased for all orientations compared with the 0° orientation (OR medial-0° = 3.21 [95% CI = 1.33–7.71] *p* = 0.009; OR 180°-0° = 4.88 [95% CI = 2.07–11.52], *p* < 0.001; OR lateral-0° = 7.73 [95% CI = 3.31–18.05] *p* < 0.001). At A3, the main effect for orientation was maintained (χ^2^ = 17.6, 3, *p* = 0.001). The 180°-0° contrast was significant (OR = 5.64 [95% CI = 2.25–14.15] *p* < 0.001) and so was the lateral-0° contrast (OR = 3.42 [95% CI = 1.34–8.69] *p* = 0.010). As at A1 the medial-0° contrast was non-significant (*p* = 0.43).

##### Within Group Effects

When running the analysis stratified by group there was a significant effect of orientation (χ^2^ = 37.25, 3, *p* < 0.001) but not age level (*p* = 0.228) within the ASD group. The likelihood of making an error was significantly increased from 0° reference for the lateral orientation (OR = 6.04, [95% CI = 3.27–11.24] *p* < 0.001), 180° orientation (OR = 4.22 [95% CI = 2.26–7.89] *p* < 0.001, and the medial orientation (OR = 2.20 [95% CI 1.15–4.20] *p* = 0.017). In comparison, the TD group had a significant change in the likelihood of making errors over the three age levels (χ^2^ = 14.2, 2, *p* < 0.001) where the likelihood of making errors at A3 was significantly reduced from A1 (A3-A1 OR = 0.41 [95% CI = 0.23–0.76] *p* = 0.005) but not from A1 to A2 (A2-A1 OR = 1.29 [95% CI = 0.79–2.10] *p* = 0.30). There was also a main effect for orientation (χ^2^ = 17.7, 3, *p* < 0.001) in the TD group. With 0° orientation as reference the likelihood of making errors were significantly higher in the 180° orientation (OR = 3.45 [95% CI = 1.72–6.91] *p* < 0.001) and in the lateral orientation (OR = 3.32 [95% CI = 1.65–6.67], *p* < 0.001). The medial orientation did not differ from 0° (OR = 1.58, *p* = 0.236).

### 3.3. Visual Imagery Task Outcomes

#### 3.3.1. Response Times

The ASD group had significantly (*F*(1, 121.3) = 20.73, *p* = 0.0001) longer RTs (higher *GMD*s) than the TD group. All the three age levels differed from one another (*F*(2, 117.6) = 27.48, *p* = 0.0001; see Table 2) with RTs gradually reducing with increasing age. An effect of orientation was also shown (*F*(3, 121.8) = 14.66, *p* = 0.0001) with the largest *GMD* being present between the 0° and 180° orientation and a minimal difference between 90° and 270° orientation (see Table 2 for specifics). Furthermore, a significant interaction was evident between age level and group (*F*(2, 117.6) = 4.26, *p* = 0.016) indicating different developmental trajectories between the groups. At A1; the ASD group had 36% higher *GMD* than the TD group (*p* = 0.001; *GMD* CI = 15%-61%); at A2 the ASD group had 50% higher *GMD* than TD (*p* = 0.0001; CI = 25–79%); and at A3 the ASD group had 19% higher *GMD* than TD (*p* = 0.034; CI = 1–39%). No significant interaction between group and orientation was shown (see Figure 2a–d).

#### 3.3.2. Error Rates

For VI, the mixed effects logistic regression with random intercept for participant ID showed that group (χ^2^ = 29.7, 1, *p* < 0.001, OR ASD-TD = 13.92 [95% CI = 5.40–35.85]), age level (χ^2^ = 38.5, 2, *p* < 0.001, OR A2-A1 = 0.98 [95% CI = 0.69–1.40]; OR A3-A1 = 0.31 [95% CI = 0.21–0.47]) and orientation (χ^2^ = 67.0, 3, *p* < 0.001, OR medial-0° = 2.34 [95% CI = 1.47–3.72], OR 180°-0° = 5.46 [95% CI = 3.48–8.55], OR lateral-0° = 1.53 [95% CI = 0.95–2.48]) predictors of the likelihood of making errors on the VI task.

##### Effects of Age Level

In the analyses stratified by age level, group was a significant predictor for the likelihood of making errors at A1 (χ^2^ = 22.5, 1, *p* < 0.001; ASD-TD OR = 43.93 [95% CI = 9.21–209.47]), A2 (χ^2^ = 17.8, 1, *p* < 0.001; ASD-TD OR = 8.87 [95% CI = 3.22–24.50]) and A3 (χ^2^ = 9.15, 1, *p* < 0.002; ASD-TD OR = 27.53 [95% CI = 3.21–236.0]). Orientation was also a significant predictor for error rates at A1 (χ^2^ = 17.5, 3, *p* < 0.001). The 180°-0° was significant (OR = 4.34 [95% CI = 2.07–9.06], *p* < 0.001) and so was the 270°-0° (OR = 1.45 [95% CI = 0.68–3.01] *p* = 0.339). The 90°-0° comparison was not significant (*p* = 0.134). Orientation remained significant at A2 (χ^2^ = 34.3, 3, *p* < 0.001) with significant differences in errors on the 90°-0° (OR = 2.95 [95% CI = 1.32–6.59], *p* = 0.008) and the 180°-0° (OR = 7.70 [95% CI = 3.51–16.86], *p* < 0.001) comparisons. The 270°-0° comparison was not significant (*p* = 0.38). At A3 orientation was still significant for the number of errors made (χ^2^ = 16.85, 3, *p* < 0.001) with significant differences in the 90°-0° (OR = 3.18 [95% CI = 1.11–9.11], *p* = 0.031) and 180°-0° (OR = 7.05 [95% CI = 2.55–19.49], *p* < 0.001) comparison. As at A2 the 270°-0° comparison was non-significant (*p* = 0.185).

##### Within Group Effects

When stratifying by group for within group analyses a significant effect was evident for age level (χ^2^ = 36.0, 2, *p* < 0.001) and orientation (χ^2^ = 40.6, 3, *p* < 0.001) within the ASD group. For age level, with A1 as reference, a decrease in the likelihood of making an error was only significant between A3 and A1 (A3–A1 OR = 0.25 [95% CI = 0.16–0.40] *p* < 0.001; A2-A1 OR = 0.74 [95% CI = 0.48–1.13] *p* = 0.16). The likelihood of errors was significant in the 180°-0° contrast (OR = 4.25 [95% CI = 2.56–7.00], *p* < 0.001) and close to significant in the 90°-0° contrast (OR = 1.66 [95% CI = 1.00–2.78] *p* < 0.051). For the TD group there were significant changes in the likelihoods of incorrect responses over age levels (χ^2^ = 9.36, 2, *p* = 0.009,) and for orientation (χ^2^ = 10.71, 3, *p* = 0.013). However, the sublevel analyses surprisingly showed increased likelihood of errors in the A2-A1 contrast (OR = 2.14 [95% CI = 1.04–4.38], *p* = 0.038) and none for the other age level contrast nor for orientation. The percentage of incorrect responses were increased from 3% at A1 to 6% at A2 (Table 3) which still reflect a high success rate at A2 in the TD group.

### 3.4. Biomechanical Effects

To answer the question if the groups use a biomechanical strategy when solving the MI task, the log RTs of the medial and lateral orientations in the MI task and 90° and 270° in the VI task was subjected to linear mixed models, one for each age level, with participant ID used as a random intercept.

#### 3.4.1. Age Level 1

Main effects were evident for group (ASD–TD: *GMD =* 18% [95% CI = 6, 32]; *F* = 8.82, 1, 545, *p* = 0.003; *p* = 0.0001); Orientation angle (Lateral/270°–Medial/90°: *GMD* = 22% [95% CI = 9, 36]; *F =* 12.06, 1, 545, *p* = 0.001); and task type (MI–VI: *GMD* = 17% [95% CI = 5, 31]; *F* = 7.7, 1, 545, *p* = 0.001). An interaction effect was further shown between group and task type (*F* = 6.98, 1, 545, *p* = 0.009) where a significant group difference was shown in the VI task (*p* = 0.0001; ASD–TD, *GMD =* 38% [95% CI = 18, 60]). There were, however, no group differences in the MI task. Further, the ASD group showed no significant difference between the MI and VI tasks whereas the TD group did; (*p* = 0.0001; MI–VI, *GMD =* 36%; [95% CI = 18, 62]). Orientation and task type did also interact (*F* = 8.39, 1, 545, *p* = 0.004) where there was a difference between the orientations on the RTs of the MI task (*p* = 0.0001; lateral–medial, *GMD =* 44% [95% CI = 22, 70]). No such difference was shown in the VI task. Further, the MI and VI task differed where the RTs were greater for MI than VI (*p* = 0.0001; MI-VI, *GMD* = 38 [95% CI = 20, 54]).

##### Within Group Differences

On the MI task there was a significant difference between the medial and lateral orientation within the ASD (lateral–medial *GMD* = 60%; Table 4) group. This difference was also evident in the TD group (lateral–medial *GMD* = 29%; Table 4). There were no differences between the corresponding orientations (270°–90°) on the VI task within any of the groups. Hence, the results from the lateral-medial MI comparison points towards a biomechanical effect being present in both groups. In comparison, the lack of difference between the 270° and 90° orientations in the VI task indicates that a visual-based strategy is being utilized. The comparison between the 90° and medial orientation and the lateral and 270° orientation within respective group showed that differences between the VI and MI were present on both the medial- 90° contrast (medial- 90° *GMD* = 21%; Table 5) and the lateral- 270° contrast (lateral- 270° *GMD* = 53%; Table 5) only in the TD group. No task differences on these orientations were evident in the ASD group.

##### Between Group Differences

There was a significant difference between the ASD and TD group in RTs in the VI task for both the 90° and 270°orientation where *GMDs* showed increased RTs in the ASD compared to the TD group (Table 6). There were no significant group differences in *GMD*s on the MI task for neither the medial or lateral orientation.

#### 3.4.2. Age Level 2

The significant group difference shown at A1 remained and had increased in magnitude at A2 (*F* = 42.40, 1, 514; *p* = 0.0001; ASD- TD; *GMD* = 49% [95% CI = 32, 68]). The main effect of orientation angle remained significant (*F* = 10.19, 1, 514, *p* = 0.002) with the lateral/270° orientations having 21% higher *GMD* than the medial/90° orientations [95% CI = 8, 37]). The MI task also had higher RTs than the VI task (*F* = 8, 03; 1, 514, *p* = 0.005; MI-VI; *GMD* = 19% [95% CI–5, 34]) similar to the effects seen at A1.

##### Within Group Differences

Within the ASD group, there where were no significant difference between the 90° and 270° orientation in the VI task nor the lateral and medial orientation in the MI task. This is showing that the biomechanical effect detected in the ASD group at A1 was no longer significant. In the TD group; however, a significant difference was evident in the MI task between the lateral and medial orientations (lateral–medial *GMD* = 41%; Table 4) indicative of a biomechanical effect. This result is strengthened by the fact that there was no difference between the 90° and 270° orientations in the VI task for the TD group indicating use of a visually based strategy (Table 4). The results from the comparison between the 90° and medial orientation and the lateral and 270° orientation within respective group were similar to that of A1. Only in the TD group were differences between the VI and MI evident on the lateral- 270° contrast (lateral–270° *GMD* = 37%; Table 5), no difference between the medial and 90° orientation was shown in the TD group. As at A1, no between tasks differences on these orientations were evident in the ASD group.

##### Between Group Differences

The groups differed significantly on all orientations (VI 90°; VI 270°; medial and lateral) where the ASD group had increased *GMDs* compared to the TD group (see Table 6 for specifics).

#### 3.4.3. Age Level 3

##### Main Effects

The ASD had significantly (*p* = 0.005; *F* = 7.833, 1, 612) higher *GMD* than the TD group (ASD-TD, *GMD = 15%,* [95% CI = 4, 28]). There was also higher RTs in the MI task compared to the VI task (*p* = 0.027; *F* = 4.93, 1, 612; *GMD =* 12% [95% CI = 1, 24]). A main effect (*p* < 0.0001; *F* = 14.31, 1, 612) was also evident for orientation where the RTs for the lateral/270° was 21% higher than medial/90° (95% CI = 10, 34]). An interaction was evident between orientation and task (*p* = 0.001; *F* = 10.36, 1, 612) where the medial and lateral orientation differed within the MI (lateral-medial *GMD =* 43% [95% CI- = 23, 66], *p* < 0.001) and the lateral orientation differed from 270° orientation between the MI and VI tasks (lateral-270° *GMD =* 32% [95% CI = 14, 53], *p* < 0.001).

##### Within Group Differences

Both the ASD and TD group showed a biomechanical effect as the *GMD* of the lateral orientation were significantly higher than the medial orientation in respective group (ASD = 55% and TD = 32% higher in the lateral orientation, Table 4). In contrast, none of the groups had a difference between the 270°and 90°roation on the VI task indicating that a visually based strategy was used here (Table 4; Figure 2a–f). In addition, both the ASD and TD groups showed a significant higher *GMD*s in the lateral compared with the 270° orientation and no significant effect when comparing the medial with the 90° orientation (Table 5; Figure 2a–f).

##### Between Group Differences

Even though *GMD*s were lower in the TD group compared to the ASD group, a group difference was only evident for the 90° orientation in the VI task (*GMD* = 26%; Table 6) in favor of lower RTs in the TD group.

## 4. Discussion

The aim of the current longitudinal study was to investigate the development of MI in children with ASD from 7 to 9 years of age and in a typically developing control group. We first established that a MI strategy was possible to detect from differences in RTs in the MI task (hand laterality judgement paradigm) from 7 years old where RTs were higher in rotations that were more biomechanically challenging. The RTs from the most biomechanically challenging rotation (i.e., lateral condition) were also greater than the RTs derived from the matched condition in the VI task (i.e., 270° rotation). A general finding was also that the RTs were higher in the ASD group compared to the TD group but both groups became faster with increasing age. A striking finding was that the ASD group was more likely to make errors compared to the TD group at all three ages and in both the MI and VI task. The biomechanical effect in the MI-task was shown at all three ages in the TD group (i.e., a consistent MI performance pattern). In the ASD group, the biomechanical effect was of considerable magnitude at A1 and A3 but did not reach significance at A2 (i.e., a less consistent MI performance pattern). Variability was high in the ASD group, specifically at A1 and A2 (Figure 2). Throughout, the TD children showed a specific difference in RTs between the lateral hand position in the MI and the 270°orientaton in the VI task, but not between any of the other rotations. The ASD group, however, showed this effect for the first time at A3.

### 4.1. Task Success and Error Rates

We initially screened the young participants before the experiment by using a criterion task in which the ability to separate between a left- and right-hand image was determined. This was done to ensure that participants were not unnecessarily exposed to a longer testing paradigm and to ensure the reliability of the results obtained. In the ASD MI literature, this does not seem to be common practice, although criterion-like tasks have occasionally been used [31]. In the present study, all the children in the TD group passed the MI and VI criterion tasks at all ages, which is similar to the effects seen in TD children at 7+ years of age [25]. In contrast, a substantial number of children from the ASD group did not pass the MI criterion task (A1 n = 5; A2 n = 4; A3 n = 2), and some further failed the VI criterion task at A1 (n = 2) and A2 (n = 2). Thus, children with ASD at these ages seem to be characterized by a developmental delay to some degree with regard to distinguishing between a right and left hand, and a correctly depicted and mirror-reversed number 2.

Previous studies that have performed analyses of MI error rates report inconsistent outcomes, where both presence [32] and absence [29,30,31] of group differences between ASD and TD have been observed in children and young adults. The present comprehensive error rate analyses from the MI task showed that the likelihood of making errors were overall higher in the ASD group than TD. This was not the case for the VI task where no group differences in the likelihood of making errors were shown. Across groups and tests, the likelihood of making errors were highest for the rotations with the most demanding positions (i.e., 180° and the lateral rotation). The same pattern of performance has been shown by others [27]. Here, we show that this effect is observed from 7 years, and it is especially evident in TD children. For the MI task, the error percentages were specifically high in the biomechanically challenging rotations for the ASD group (see Table 3) which also is shown in the OR analyses where, for example, the OR for the ASD children to make an error in the lateral condition was 6.71 when compared to the 0° condition. As a reference, the TD group’s OR for the same comparison was 3.32. Furthermore, the ASD group did not show a significant reduction in errors over the age levels indicating a maintenance of difficulties in solving the MI task. In contrast, the likelihood of making errors in the VI task was reduced with age in the ASD group. This can be taken as evidence for delays in the development of motor representations in ASD, likely relating to the motor planning and performance disturbances shown in previous research including the same children [20]. However, both tasks were more difficult for the children with ASD than controls at all ages. The reduced likelihood to make errors in the VI task with increasing age suggests that the ability to perform visually based mental rotations improved with maturation. The greater error rates for the difficult rotations in the ASD group poses a challenge to the current study as it reduces the available RT estimates for the biomechanical effect analyses and thus could contribute to increased uncertainty in these results. Other studies investigating MI in ASD have included samples with older children, at greater age ranges, and with higher verbal and full-scale IQ [29,31,32] than the current study. How these parameters affect the comparability between outcomes from these studies and the current one is unclear, but the higher error rates seen here could, at least in part, be due to the younger ages and comparatively lower developmental level of the participating children with ASD.

### 4.2. Response Times and the Biomechanical Effect

Previous studies have shown varying occurrence of and stability in the expression of MI in ASD [29,31,32]. The results of the current study showed that a biomechanical effect was present in the ASD group at A1 and A3. However, large variability in these RTs and elevated error rates in the lateral rotation within the ASD group lead to uncertainty in the stability of the results. As an example, at A1 the *GMD* in the ASD group was 60% (95% CI = 21,111) to be compared with the TD group who had a *GMD* of 29% (95% CI = 8, 55). The absence of a significant difference for ASD at A2 can have many causes. One possibility is that the increased number of errors may have led to reduced power in the analysis for that age level. Another possibility is higher within-group variability. Still, with a 95% CI range from -6 to 76 (*GMD* = 29%), the variability does not seem to be that different from A1 and A3. Given the relatively high *GMD* at A2 paired with the lack of difference between the 90° and 270° orientations in the A2 VI condition, and the relatively stable MI CIs over age there seem to be a power issue in detecting potential effects at A2. As there were no difference between the 90° and 270° rotation within any of the groups at any of the age levels (Table 4), this offers support to the interpretation that different strategies were being utilized in the different tasks by both groups. Chen and colleagues [29] showed a presence of a biomechanical effect, although RTs were higher in ASD than TD controls. This paired with processing speed for a VI based task being similar between the groups led the authors to conclude that MI was present in their ASD sample but inefficient. Similar results have been presented with intact biomechanical effects in ASD on a, to the current study, comparable MI task but with intrusion effects of incongruent actual hand postures with MI stimuli in ASD indicating less efficient MI [31]. In keeping with Chen et al. [29], we also showed a general tendency of higher RTs for the ASD group compared with TD, regardless of test type, but also for the MI task compared to the VI task. Our follow-up analyses revealed that the TD group primarily was driving the difference in RTs between the MI and VI tasks. This was particularly evident at A1 and A2, where the ASD group showed no difference between the VI and MI task rotations as opposed to the TD group. The ASD group showed a task sensitivity first at A3, with significantly increased *GMD* in the lateral compared to the 270° rotation (Table 5). Hence, the significant *GMD*s between MI and VI were primarily related to differences in the lateral/270° conditions being specifically manifest in the TD group. Other studies [29,31] typically show greater RT differences between MI and VI tasks using both symbols [31] and abstract stimuli [29]. Noteworthy is that these studies have included older children than our sample. Thus, it is possible that the similarities in RTs between the MI and VI tasks in the current study reflect a not yet automatized representation of numeric symbols in turn affecting mental processing speed of the VI stimuli negatively. The VI task seems especially difficult to perform for the ASD group given the high error rates. It is suggested that future research should consider if and how performance relates to cognitive capacities, including working memory.

Limitations with the present study include the relatively low number of participants and the differences between the TD and ASD groups regarding gender distribution, intellectual function and handedness. The limited sample size and associated variability may reduce the possibility to detect potential group differences. We tried to overcome individual variation affecting the outcomes too greatly by using mixed model statistics with each participant having its own unique intercept. However, it is important to consider these limitations when interpreting the results. Still, validity of the results is increased by the coherent age span and the longitudinal design, providing a rare developmental perspective regarding MI ability in ASD and TD children.

Taken together, our longitudinal study shows higher inability for the ASD group to perform the MI task throughout the study period. This is manifested by difficulties in passing the criterion task, especially at A1, and in increased error rates as compared to TD children. The biomechanical effect, as defined as a significant difference between the medial and lateral rotation in the MI task, was present in both groups, although less stable in ASD compared with TD over age. Increased error rates in the MI task for the most biomechanically challenging positions mimics that of the TD group, although heavily inflated in the ASD group compared to controls. This is different from the pattern shown on the VI task where the error rate for the 90° and 270° is more similar to one another (Table 3). Thus, the lateral MI condition was more difficult than the VI task for the ASD group, which might be indicative of an immature ability to imagine body positions. At A3, error rates remained high for the ASD group. Although a biomechanical effect was shown at A1 and A3 in the ASD group error rates remained high throughout. Hence, the children with ASD displayed a protracted MI development as compared to TD children as reflected both by the error rates and RTs. It is also possible that this ability, at least for some individuals, may be impaired. Even though only backhand images were used in the present study, the high error rates in the ASD group suggest that the task is very demanding for this group of children. It is difficult to know if the included children with ASD show a developmental delay or deviation in the abilities measured. Hence, there is a need for continued longitudinal studies also including older children. Furthermore, how mental rotations of hands are related to actual planning of manual actions needs to be further evaluated in this group of children to verify the impact MI may have on actual movements.

## 5. Conclusions

The present results show that children with ASD have problems with mentally rotating visual images and especially with mentally rotating depicted body parts (right and left hands). There was, however, a wide performance variability in the ASD group where initially five children, at the age of seven, failed the MI criterion task. For all age levels and for both the motor imagery and visual imagery tasks, the error rates were significantly higher and RTs longer for the ASD group compared to TD. It is concluded that the ability to form mental representations and especially motor representations, is weakened and/or its development protracted in children with ASD. This inability has consequences for the formation of ideas of how body parts will move during action and could lead to a wide range of problems associated with motor disturbances. Thus, children with ASD may benefit from additional support when learning new motor actions.

## Figures and Tables

**Table 1:** Intellectual function at A1 by group.

	ASD (N = 14)			TD (N = 17)		
	Mean ± S.D.	Median	Range	Mean ± S.D.	Median	Range
FSIQ	82 ± 15.3	86	54–110	106 ± 9.4	104	93–126
VeI	83 ± 17.0	81	48–106	108 ± 7.0	106	100–127
FI	93 ± 16.0	94	72–126	102 ± 9.6	100	88–118
WMI	84 ± 15.8	88	65–115	103 ± 10.5	103	88–122

Note: FSIQ = Full Scale Intelligence Quotient; VeI = Verbal Index; FI = Fluid Index; WMI = Working Memory Index (all from WISC-V); S.D. = standard deviation; ASD = autism spectrum disorder; TD = typically developing.

**Table 2:** Main effects and factorial contrasts organized by test type.

Test	Factor	Contrast	*GMD* %	95% CI	*F*	df	*p*
MI	Group	-	-	-	3.90	1; 113.0	0.051
		ASD–TD	17	−0.1; 37	-	-	0.051
	Age level	-	-	-	20.33	2; 108.5	0.0001
		A1–A2	25	5; 48	-	-	0.008
		A1–A3	53	29; 81	-	-	0.0001
		A2–A3	23	7; 40	-	-	0.001
	Orientation	-	-	-	9.42	2; 111.8	0.0001
		0°–medial	−9	−32; 22	-	-	0.955
		0°–180°	−40	−56; −20	-	-	0.0001
		0°–lateral	−35	−52, −12	-	-	0.001
		medial–180°	−35	−51; −12	-	-	0.001
		medial–lateral	−28	−47; −4	-	-	0.020
		180°–lateral	9	19; 48	-	-	0.960
VI	Group	-	-	-	20.73	1; 121.33	0.0001
		ASD–TD	34	18; 53	-	-	0.0001
	Age level	-	-	-	27.48	2; 117.61	0.009
		A1–A2	7	6; 23	-	-	0.521
		A1–A3	35	20; 52	-	-	0.0001
		A2–A3	26	14; 39	-	-	0.0001
	Orientation	-	-	-	14.66	3; 121.76	0.0001
		0°–90°	−20	−37, 1	-	-	0.014
		0°–180°	−45	−57; −30	-	-	0.0001
		0°–270°	−21	−38; 0,5	-	-	0.058
		90°–180°	−31	−46; −17	-	-	0.001
		90°–270°	−1	−22; 26	-	-	1.0

MI = motor imagery task; VI = visual imagery task; *GMD* = geometrical mean difference; 95% CI = confidence interval of the *GMD*s; df = degrees of freedom; *p* = alpha estimate; A1 = age level 1; A2 = age level 2; A3 = age level 3.

**Table 3:** Descriptive statistics of the percentage and number of correct responses, RTs and LnRTs and associated data presented by group, orientation, overall orientations, and age level for the MI and VI test.

Task	Group	Orient- Ation	n cor. (% cor.)	Mdn RT *	IQR RT *	*M* LnRT	95% CI LnRT	n cor. (% cor.)	Mdn RT *	IQR RT *	*M* LnRT	95% CI LnRT	n cor. (% cor.)	Mdn RT *	IQR RT *	*M* LnRT	95% CI LnRT
MI	ASD	0°	48 (89)	1940	1426	7.65	7.48–7.83	43 (90)	1660	1012	7.58	7.38–7.77	53 (84)	1346	778	7.21	7.07–7.35
		Medial	42 (78)	1816	1461	7.57	7.41–7.73	36 (75)	2333	2012	7.8	7.59–8.01	52 (81)	1383	873	7.28	7.12–7.43
		180°	34 (63)	3626	3661	8.09	7.81–8.38	34 (71)	3885	5363	8.2	7.91–8.49	44 (70)	2431	1837	7.81	7.60–8.01
		Lateral	33 (61)	2780	4212	8.03	7.76–8.31	25 (52)	3268	5443	8.05	7.70–8.41	42 (68)	2163	1871	7.71	7.50–7.39
		Overall	157 (73)	2281	2603	7.8	7.69–7.92	138 (78)	2365	2713	7.87	7.74–8.01	191 (76)	1645	1434	7.48	7.38–7.57
	TD	0°	95 (93)	1947	1322	7.55	7.45–7.66	98 (96)	1342	998	7.29	7.19–7.39	101 (99)	1075	554	7.05	6.97–7.13
		Medial	96 (94)	1853	1466	7.65	7.53–7.77	92 (90)	1441	890	7.58	7.28–7.48	100 (98)	1304	655	7.21	7.17–7.36
		180°	94 (92)	2491	2254	7.99	7.86–8.11	87 (85)	2491	1733	7.8	7.69–7.90	91 (89)	1904	1198	7.63	7.53–7.73
		Lateral	87 (85)	2311	3697	7.55	7.75–8.07	87 (85)	1910	2686	7.73	7.57–7.89	99 (97)	1681	1390	7.91	7.41–7.68
		Overall	372 (91)	2135	1833	7.77	7.71–7.84	364 (89)	2274	1471	7.54	7.47–7.60	391 (96)	1514	1041	7.37	7.31–7.42
VI	ASD	0°	52 (72)	1684	1244	7.52	7.35–7.70	48 (80)	1607	1459	7.61	7.36–7.85	66 (92)	1091	971	7.21	7.05–7.36
		90°	46 (64)	2191	1627	7.77	7.59–7.96	43 (72)	2265	1358	7.74	7.56–7.91	64 (86)	1555	1217	7.46	7.31–7.61
		180°	39 (54)	3007	4293	8.15	7.89–8.40	29 (48)	2855	2010	8.02	7.75–8.29	49 (68)	2455	1462	7.77	7.58–7.95
		270°	49 (68)	2234	2676	7.81	7.62–8.00	47 (78)	2372	1299	7.82	7.63–8.00	67 (89)	1448	991	7.41	7.25–7.58
		Overall	186 (35)	2175	2129	7.79	7.69–7.90	167 (69)	2274	1789	7.77	7.66–7.88	246 (84)	1514	1495	7.44	7.36–7.52
	TD	0°	102 (0)	1286	944	7.25	7.15–7.36	102 (100)	1099	661	7.05	6.97–7.13	102 (100)	1064	565	7.03	6.96–7.11
		90°	100 (98)	1509	1146	7.46	7.36–7.55	93 (91)	1367	839	7.3	7.19–7.42	97 (94)	1202	667	7.19	7.09–7.28
		180°	92 (90)	2411	2059	7.86	7.76–7.97	88 (86)	2110	1495	7.78	7.66–7.90	100 (98)	1765	1162	7.6	7.50–7.70
		270°	100 (98)	1585	1052	7.49	7.39–7.58	99 (97)	1558	1225	7.41	7.30–7.51	99 (97)	1341	646	7.29	7.17–7.41
		Overall	394 (97)	1704	1323	7.51	7.45–7.56	382 (94)	1468	1105	7.37	7.31–7.43	398 (97)	1325	781	7.28	7.22–7.33

MI = motor imagery task; VI = visual imagery task; n cor. = number correct responses; % cor. = percentage correct responses; 95% CI = confidence interval; IQR = inter quartile range; * = millisecond; RT = response time; LnRT = logarithmed RT; M = mean; p = alpha estimate; A1 = age level 1; A2 = age level 2; A3 = age level 3.

**Table 4:** Within group effects of the biomechanical (lateral-medial) and visual mental rotation (270°–90°) contrasts.

	ASD						TD					
	Lateral–Medial			270°–90°			Lateral–Medial			270°–90°		
	*GMD*%	95% CI	*p*	*GMD*%	95% CI	*p*	*GMD*%	95% CI	*p*	*GMD*%	95% CI	*p*
A1	60	21, 111	0.001	4	−19, 33	0.761	29	8, 55	0.005	3	−13, 22	0.753
A2	29	−6, 76	0.118	8	−16, 40	0.556	41	18, 69	0.0001	11	−7, 32	0.243
A3	55	21, 98	0.001	−4	−22, 18	0.667	32	12, 56	0.001	11	−6, 31	0.225

MI = motor imagery task; VI = visual imagery task; *GMD* = geometrical mean difference; 95% CI = confidence interval of the *GMD*s; *p* = alpha estimate; A1 = age level 1; A2 = age level 2; A3 = age level 3.

**Table 5:** Within group effects of between test comparisons (medial—90°) and (lateral—270°) contrasts.

	ASD						TD					
	Medial—90°			Lateral—270°			Medial—90°			Lateral—270°		
	*GMD*%	95% CI	*p*	*GMD*%	95% CI	*p*	*GMD*%	95% CI	*p*	*GMD*%	95% CI	*p*
A1	−19	−37, 5	0.115	25	−5, 64	0.107	21	2, 44	0.027	53	28, 82	0.0001
A2	6	−19, 40	0.661	30	−6, 71	0.123	8	−10, 29	0.400	37	15, 64	0.001
A3	−17	−35, −8	0.109	35	7, 71	0.012	8	−19, 28	0.358	29	9, 53	0.003

*GMD* = geometrical mean difference; 95% CI = confidence interval of the *GMD*s; *p* = alpha estimate; A1 = age level 1; A2 = age level 2; A3 = age level 3.

**Table 6:** Between group effects of the medial, lateral, 90° and 270° orientations.

ASD-TD												
	MI: Medial			MI: Lateral			VI: 90°			VI: 270°		
	*GMD*%	95% CI	*p*	*GMD*%	95% CI	*p*	*GMD%*	95% CI	*p*	*GMD%*	95% CI	*p*
A1	−8	−26, 15	0.448	13	−12, 45	0.325	37	11, 70	0.004	38	12, 71	0.002
A2	52	20, 93	0.001	39	5, 83	0.020	54	23, 93	0.0001	50	21, 86	0.0001
A3	6	−13, 31	0.554	20	−3, 50	0.098	26	4, 52	0.018	11	−8, 33	0.296

*GMD* = geometrical mean difference; 95% CI = confidence interval of the *GMD*s; *p* = alpha estimate; A1 = age level 1; A2 = age level 2; A3 = age level 3.

## Data Availability

Data available upon request to the corresponding author.
